# Whole Genome Comparison Reveals High Levels of Inbreeding and Strain Redundancy Across the Spectrum of Commercial Wine Strains of *Saccharomyces cerevisiae*

**DOI:** 10.1534/g3.115.025692

**Published:** 2016-02-11

**Authors:** Anthony R. Borneman, Angus H. Forgan, Radka Kolouchova, James A. Fraser, Simon A. Schmidt

**Affiliations:** * The Australian Wine Research Institute, PO Box 197, Glen Osmond, South Australia 5064, Australia; † Department of Genetics and Evolution, University of Adelaide, South Australia 5000, Australia; ‡ Australian Infectious Diseases Research Centre and School of Chemistry and Molecular Biosciences, The University of Queensland, Brisbane, Queensland 4072, Australia

**Keywords:** genome sequencing, industrial yeast, comparative genomics, fermentation

## Abstract

Humans have been consuming wines for more than 7000 yr . For most of this time, fermentations were presumably performed by strains of *Saccharomyces cerevisiae* that naturally found their way into the fermenting must . In contrast, most commercial wines are now produced by inoculation with pure yeast monocultures, ensuring consistent, reliable and reproducible fermentations, and there are now hundreds of these yeast starter cultures commercially available. In order to thoroughly investigate the genetic diversity that has been captured by over 50 yr of commercial wine yeast development and domestication, whole genome sequencing has been performed on 212 strains of *S. cerevisiae*, including 119 commercial wine and brewing starter strains, and wine isolates from across seven decades. Comparative genomic analysis indicates that, despite their large numbers, commercial strains, and wine strains in general, are extremely similar genetically, possessing all of the hallmarks of a population bottle-neck, and high levels of inbreeding. In addition, many commercial strains from multiple suppliers are nearly genetically identical, suggesting that the limits of effective genetic variation within this genetically narrow group may be approaching saturation.

Humans have been producing and consuming wines for more than 7000 yr, making wine one of the first processed agricultural products (Sicard and Legras 2011). Until the middle of the 20th century, winemaking relied on naturally occurring yeasts to complete the fermentation process. However, spontaneous fermentations such as these, produced inconsistent results from vintage to vintage and, due to their protracted fermentation times, were often vulnerable to spoilage by undesirable yeast and/or bacteria.

One of the most significant technological advances in winemaking was the introduction of pure starter strains of the major wine yeast, *Saccharomyces cerevisiae*, in the 1950s, with the first commercial active dried starters being released in 1965. Most commercial wine fermentations are now inoculated with high numbers of these selected strains directly after the grapes are crushed, to ensure consistent, reliable and reproducible fermentations (Heard and Fleet 1985; Henick-Kling *et al.* 1998). Since their introduction, hundreds of strains of *S. cerevisiae* have been have been developed into a wide variety of commercial starter cultures.

Genome sequencing has shown that, in general, vineyard and wine strains form a phylogenetically related group (Fay and Benavides 2005; Liti *et al.* 2009; Borneman *et al.* 2011). Recently, this group has also been shown to contain strains from Mediterranean oaks, which may be the historical progenitor of “domesticated” wine yeasts (Almeida *et al.* 2015). Furthermore, strains isolated from wineries or vineyards outside of Europe are unrelated to “indigenous” *S. cerevisiae* strains, except in cases of close proximity to winemaking environs (Hyma and Fay 2013). This suggests that European wine strains have accompanied the migration of winemaking around the globe, and are maintained as distinct populations through phenotypic selection (Fay *et al.* 2004; Warringer *et al.* 2011; Clowers *et al.* 2015a). Interestingly, despite their common geographic origins, and roles in the production of alcoholic beverages, wine strains are also genetically distinct from *S. cerevisiae* strains used for brewing (Borneman *et al.* 2011; Dunn *et al.* 2012).

In order to investigate the genetic diversity that has been captured by over 50 yr of commercial wine yeast development, whole genome sequencing was performed on 212 strains of *S. cerevisiae*, including 106 commercial wine starter strains from nine different commercial yeast suppliers. In addition to the wine yeast strains, 13 commercially available brewing strains were also sequenced to compare general features of the two industrial groups. Comparative genomic analysis shows that, despite their large numbers, commercial strains, and wine strains in general, remain genetically similar, with a population bottle-neck and/or high levels of inbreeding apparent between isolates. In addition, commercial strains from multiple suppliers are often genetically identical, suggesting that the limits of effective natural variation within this group may have been reached.

## Materials and Methods

### DNA isolation, extraction, and sequencing

Noncommercial wine yeast isolates were obtained from The Australian Wine Research Institute (AWRI) Microorganisms Culture Collection. Commercial strains were obtained as purified strains from the manufacturer, or were sourced as active-dried yeast preparations.

For strains from the AWRI culture collection, samples were grown overnight at 28° in YPD. For the commercial strains obtained from freeze-dried packets, 5 g of active dry yeast was rehydrated in 50 ml of water (38°) for 20–40 min to obtain an homogenous suspension of rehydrated yeast; 25 µl of rehydrated yeast was then inoculated into 5 ml of YPD and grown overnight at 28°. For both culture types, 1.5 ml of the overnight culture was used for DNA extraction (Gentra Puregene Yeast/Bact kit, Qiagen). For strains from the White labs (http://www.whitelabs.com), and WYeast collections (https://www.wyeastlab.com), strains were grown overnight in YPD at 30° with shaking. Washed cell pellets were frozen and lyophilized, and DNA extracted via the CTAB extraction method as described (Pitkin *et al.* 1996).

Genomic libraries for AWRI strains were prepared using the Nextera XT platform (Illumina), and sequenced using Illumina Miseq, paired-end 300 bp chemistry (Ramaciottti Centre for Functional Genomics, University of New South Wales, Australia). White Labs and WYeast strains were sequenced using paired-end 100 bp chemistry (BGI).

### Sequence processing and reference-based alignment

An extended *Saccharomyces sensu stricto* reference sequence was assembled from existing genomic sequences for *S. cerevisiae* (Goffeau *et al.* 1996), *S. paradoxus*, *S. mikatae*, *S. kudriavzevii*, *S. uvarum* (Scannell *et al.* 2011), and *S. arboricolus* (Liti *et al.* 2013). As a *de novo* assembled genome was not available for *S. eubayanus*, the non-*S. cerevisiae* contribution of the *S. pastorianus* genome (*S. cerevisiae–S. eubayanus* hybrid) was used as a proxy (Nakao *et al.* 2009). In addition to these reference genomes, 26 pan-genomic segments from *S. cerevisiae* were included in order to track the presence of these elements (Supplemental Material, File S1), which included key industry-associated elements from wine, brewing, biofuel, and sake yeasts (Ness and Aigle 1995; p. 6 in Hall and Dietrich 2007; Novo *et al.* 2009; Argueso *et al.* 2009; Borneman *et al.* 2011; Akao *et al.* 2011).

Raw sequence data were quality trimmed [trimmomatic v0.22 (Bolger *et al.* 2014); TRAILING:20 MINLEN:50], and aligned to the extended *Saccharomyces sensu stricto* clade using novoalign (v3.02.12; -n 300 -i PE 100-1000 -o SAM; http://www.novocraft.com/) and converted to sorted .bam format using samtools (v1.2; Li *et al.* 2009). Single nucleotide variation between the reference genome and each strain was performed using Varscan (v2.3.8;–min-avg-qual 0–min-var-frequation 0.3–min-coverage 10; Koboldt *et al.* 2012), and this information was used to alter a coverage masked-reference sequence to reflect these differences using custom python scripts. Maximum-likelihood phylogenies were then produced from these altered reference sequences using Seaview (v4.4.2; -phyml; Gouy *et al.* 2010).

### Genome analysis

Copy number analysis was performed on the per-base coverage information included in the output of samtools mileup (v1.2; Li *et al.* 2009) with a custom python script used to apply smoothing via a 10-kb sliding window, with a 5-kb step. Results were presented relative to the mean coverage of all windows containing at least 10 reads.

Heterozygosity levels were calculated by recording the total number of heterozygous and homozygous single nucleotide polymorphisms (SNPs) called for each strain relative to the reference using Varscan (Koboldt *et al.* 2012). Results were smoothed using a 10-kb sliding window, with a 5-kb step via custom python scripts.

Identity-by-state (IBS) analysis was performed by recording the total number of shared alleles between all pairwise combinations of strains across all genomic locations in which a SNP was recorded in at least one strain, and for which data were missing in less than two strains. IBS state 2 (IBS2) represents identical diploid genotypes (*e.g.*, AA:AA, AT:AT), IBS1 loci share one allele (*e.g.*, AA:AT; AC:AT), while IBS0 loci are completely different (*e.g.*, AA:TT; AT:CG). Results were then smoothed using a 50-kb sliding window, with a 25-kb step, with individual windows further categorized according to four genomic states IBS2 (< 5% IBS1, < 1% IBS0), IBS2|1 (≥ 5% IBS1, < 1% IBS0), IBS2|1|0 (≥ 5% IBS1, ≥1 % IBS0) and IBS2|0 (< 5% IBS1, ≥ 1% IBS0).

### Data availability

All mapped sequence data have been deposited in the NCBI short read archive under the accession number SRP066835 (BioProject accession PRJNA303109). All AWRI-designated strains sequenced in this study are available from the AWRI Wine Microorganism Culture Collection, while the WL- and WY-designated strains are available from Dr. James A. Fraser.

## Results and Discussion

Whole-genome sequence data were generated for 212 *S. cerevisiae* strains, of which 106 are commercially available strains from nine different yeast supply companies. In addition to these 212 strains, another 24, from a variety of sources, and for which existing whole-genome sequence was available, were used for comparison purposes, resulting in a total of 236 strains for which analysis was performed (Table 1).

**Table 1:** Yeast strains sequenced in this study

Strain	Other name	Origin	Clade
AWRI81		Australia 1940; sherry	Vin7
AWRI170		Australia 1947; champagne	Wine
AWRI213		Australia 1949; wine	Wine
AWRI228		Australia 1949; grapes	Wine
AWRI266		Pre1937	Wine
AWRI292		Australia 1949; sherry	Vin7
AWRI722		Australia	Vin7
AWRI723		Australia	Vin7
AWRI724		Canada 1961; Tokay	Wine
AWRI735		Switzerland; pre1967	Other
AWRI739		England, pre1967; apple skin	Other
AWRI740		Germany; pre1968	Vin7
AWRI763		Australia 1971; wine	Vin7
AWRI766	WE1	South Africa; pre1971	Wine
AWRI767	WE14	South Africa; pre1971	Vin7
AWRI778		Australia 1973; wine	Vin7
AWRI792	Isolate of WE1	New Zealand, pre1975	Wine
AWRI793	Isolate of AWRI792 (WE1)	New Zealand, pre1975	Wine
AWRI795	Isolate of WE14	New Zealand, pre1975	Vin7
AWRI796*^a^*	AWRI796	Maurivin; South Africa; pre1975	Wine
AWRI814		Australia 1964; wine	Wine
AWRI833		Australia 1979; wine	Wine
AWRI834		Australia 1979; champagne	Wine
AWRI838			PdM
AWRI858*^a^*	Oenoferm	Erbsloh	Wine
AWRI896			Wine
AWRI931	UCD C-14		Other
AWRI932	UCD C-237	USA, 1939; grapes	Wine
AWRI934	UCD 48-41		Other
AWRI935	UCD C-9	USA, 1940; wine	Vin7
AWRI937	UCD 55-97		Wine
AWRI939	UCD 62-9	1962; Sake	Other
AWRI947			Wine
AWRI971			Wine
AWRI1001		France	PdM
AWRI1017		Australia	Wine
AWRI1077	UCD 513	Distilling	Wine
AWRI1078	UCD 514	Wine	Wine
AWRI1082	NCYC 761	Nigeria 1973; palm wine	Other
AWRI1083	NCYC 738	England 1972; brewery	Other
AWRI1427*^a^*	Lalvin WSK 27	Lallemand	Wine
AWRI1428*^a^*	Uvaferm PM	Lallemand	PdM
AWRI1429*^a^*	Lalvin DV10	Lallemand; France	PdM
AWRI1430*^a^*	EnofermAssmunshansen	Lallemand	Wine
AWRI1431*^a^*	Lalvin W15	Lallemand; Switzerland; wine	Wine
AWRI1432*^a^*	Vin7	Anchor Yeast	Vin7
AWRI1435*^a^*	Oenoferm Klosterneuberg	Erbsloh	Wine
AWRI1436*^a^*	Lalvin S6U	Lallemand	Vin7
AWRI1451*^a^*	Levuline CHP	Lallemand; France	PdM
AWRI1474		Australia, 2003	Vin7
AWRI1482		Australia 2004; wine	Wine
AWRI1483*^a^*	Lalvin ICV D254	Lallemand; France; wine	Wine
AWRI1484*^a^*	Lalvin Rhone L2226	Lallemand; France; vineyard	Wine
AWRI1485*^a^*	Lalvin RC212	Lallemand; France; wine	Wine
AWRI1486*^a^*	Lalvin BM45	Lallemand	Wine
AWRI1487*^a^*	Lalvin Rhone L2056	Lallemand	Wine
AWRI1488*^a^*	Lalvin ICV D47	Lallemand; France; wine	Wine
AWRI1489*^a^*	Levuline BRG	Lallemand	Wine
AWRI1490*^a^*	Lalvin Rhone L2323	Lallemand	Wine
AWRI1491*^a^*	Uvaferm BDX	Lallemand; France	Wine
AWRI1492*^a^*	Enoferm CSM	Lallemand; France	Wine
AWRI1493*^a^*	Lalvin 71B	Lallemand	Other
AWRI1494*^a^*	Enoferm M2	Lallemand	Wine
AWRI1495*^a^*	Uvaferm CM 522	Lallemand	Wine
AWRI1496*^a^*	Enoferm SYRAH	Lallemand; France	Wine
AWRI1497*^a^*	Lalvin T73	Lallemand; Spain	Wine
AWRI1498*^a^*	Lalvin EC1118	Lallemand	PdM
AWRI1501	*S. cerevisiae* × *S. paradoxus*	AWRI	PdM
AWRI1502*^a^*	AWRI Fusion	Maurivin	PdM
AWRI1503*^a^*	AWRI1503	Maurivin	PdM
AWRI1504	*S. cerevisiae* × *S. mikatae*	AWRI	PdM
AWRI1505*^a^*	Cerebray	Maurivin	PdM
AWRI1537*^a^*	Vin13	Anchor Yeast	Wine
AWRI1575*^a^*	N96	Anchor Yeast	PdM
AWRI1616*^a^*	PDM	Maurivin	PdM
AWRI1625*^a^*	Levuline C19	Oenofrance	PdM
AWRI1629*^a^*	Lalvin BA11	Lallemand; Spain	Wine
AWRI1638*^a^*	Platinum	Maurivin	PdM
AWRI1639*^a^*	Distinction	Maurivin	PdM
AWRI1642*^a^*	Advantage	Maurivin	PdM
AWRI1686		Australia, 1988	Wine
AWRI1688*^a^*	Zymaflore VL3c	Laffort	Wine
AWRI1697	Isolate of N96	Australia, 2009	PdM
AWRI1705		Germany, pre1981	Wine
AWRI1706		Australia, pre1981; wine	Wine
AWRI1709			Wine
AWRI1712		Australia; wine	Wine
AWRI1714		Australia 1981; wine	Wine
AWRI1716		Australia, pre1980	Wine
AWRI1719		Australia 1981; wine	Wine
AWRI1722	CBS 7045	wine	Other
AWRI1724		Australia; wine	Wine
AWRI1727			Wine
AWRI1729			Other
AWRI1736		Australia; wine	Wine
AWRI1742		Australia; wine	Wine
AWRI1743		Australia; wine	Wine
AWRI1744		France; wine	Wine
AWRI1754		France	Wine
AWRI1756		France; wine	Wine
AWRI1757		France	Wine
AWRI1758		France	Wine
AWRI1759		France	Wine
AWRI1760		France	Wine
AWRI1761		France; 1982	Wine
AWRI1762		France; 1982	PdM
AWRI1775		Australia; sherry	PdM
AWRI1776			Wine
AWRI1778			Wine
AWRI1781		France	Wine
AWRI1782		France	Wine
AWRI1784		France	Wine
AWRI1787			Wine
AWRI1795			PdM
AWRI1796		Australia 1984; wine	PdM
AWRI1833*^a^*	Uvaferm 43	Lallemand	Wine
AWRI1899*^a^*	Fermichamp	DSM; France	Vin7
AWRI1901		France	Wine
AWRI1902		France	PdM
AWRI1910	NCYC 738	England 1972; brewery	Other
AWRI1912		France	PdM
AWRI1915		Italy	Vin7
AWRI1918		Germany	Vin7
AWRI1921		South Africa	Wine
AWRI1939		Australia, 1989	PdM
AWRI1942	UCD 713	France; wine	Wine
AWRI1946	UCD 725	France; wine	Wine
AWRI1950*^a^*	Oenoferm LWE317-28	Erbsloh	Wine
AWRI1962		Australia, 1990	PdM
AWRI2006	IOC B 2000	Institut Oenologique de Champagne	Wine
AWRI2013	Blastocel Grand Cru		Wine
AWRI2077*^a^*	Lalvin ICV D21	Lallemand; France; wine; 1999	Wine
AWRI2078*^a^*	Lalvin CY3079	Lallemand; France	Wine
AWRI2079*^a^*	Enoferm T306	Lallemand; Australia; wine	Wine
AWRI2255*^a^*	Uvaferm HPS	Lallemand	Wine
AWRI2260*^a^*	Lalvin QA23	Lallemand	PdM
AWRI2308		Australia 2011; wine	Wine
AWRI2340	IOC 18-2007	Institut Oenologique de Champagne	PdM
AWRI2768		Australia 2013; wine	Wine
AWRI2776*^a^*	NT116	Anchor Yeast	PdM
AWRI2848*^a^*	Actiflore B0213	Laffort	Wine
AWRI2849*^a^*	Actiflore F33	Laffort	Wine
AWRI2850*^a^*	Actiflore RMS2	Laffort	PdM
AWRI2851*^a^*	Actiflore Rose	Laffort	Wine
AWRI2852*^a^*	Zymaflore F15	Laffort; France	Wine
AWRI2853*^a^*	Zymaflore F83	Laffort; Italy	Wine
AWRI2854*^a^*	Zymaflore FX10	Laffort	Wine
AWRI2855*^a^*	Zymaflore RB2	Laffort; France	Wine
AWRI2856*^a^*	Zymaflore RX60	Laffort	Wine
AWRI2858*^a^*	Zymaflore VL1	Laffort	Wine
AWRI2859*^a^*	Zymaflore VL2	Laffort; France	Wine
AWRI2860*^a^*	Zymaflore X16	Laffort	Wine
AWRI2861*^a^*	Zymaflore X5	Laffort	Wine
AWRI2863*^a^*	Collection Cepage Chardonnay	Oenobrands	Wine
AWRI2864*^a^*	Collection Cepage Merlot	Oenobrands; France	Wine
AWRI2865*^a^*	Collection Cepage Pinot	Oenobrands	Other
AWRI2866*^a^*	Collection Cepage Sauvignon Blanc	Oenobrands; France	Wine
AWRI2867*^a^*	Collection Cepage Syrah	Oenobrands	Wine
AWRI2868*^a^*	Fermichamp	Oenobrands	Vin7
AWRI2869*^a^*	Fermicru 4F9	Oenobrands; France	PdM
AWRI2870*^a^*	Fermicru AR2	Oenobrands; France	Wine
AWRI2871*^a^*	Fermicru LS2	Oenobrands; France	PdM
AWRI2872*^a^*	Fermicru LVCB	Oenobrands; Chile	PdM
AWRI2873*^a^*	Fermicru Rose	Oenobrands	PdM
AWRI2874*^a^*	Fermicru VR5	Oenobrands; France	Wine
AWRI2875*^a^*	Fermicru XL	Oenobrands	Wine
AWRI2876*^a^*	Fermirouge	Oenobrands; France	Wine
AWRI2877*^a^*	Fermivin	Oenobrands; France	Wine
AWRI2878*^a^*	NT112	Anchor Yeast	PdM
AWRI2879*^a^*	NT202	Anchor Yeast	PdM
AWRI2880*^a^*	NT50	Anchor Yeast	PdM
AWRI2881*^a^*	Vin2000	Anchor Yeast	PdM
AWRI2882*^a^*	WE14	Anchor Yeast	Wine
AWRI2895*^a^*	VINTAGE RED	Enartis	Wine
AWRI2896*^a^*	REDFRUIT	Enartis	Wine
AWRI2897*^a^*	ES 181	Enartis	Wine
AWRI2898*^a^*	ES 454	Enartis	Wine
AWRI2899*^a^*	Aroma White	Enartis	Wine
AWRI2900*^a^*	ES 123	Enartis	Wine
AWRI2901*^a^*	Vintage White	Enartis	PdM
AWRI2902*^a^*	Perlage	Enartis	PdM
AWRI2903*^a^*	EZFERM	Enartis	Wine
AWRI2904*^a^*	EZFERM 44	Enartis	Vin7
AWRI2905*^a^*	TOP 15	Enartis	PdM
AWRI2906*^a^*	TOP FLORAL	Enartis	Other
AWRI2907*^a^*	Maurivin B	Maurivin	Wine
AWRI2908*^a^*	Maurivin BP725	Maurivin; France	Wine
AWRI2909*^a^*	Maurivin Cru Blanc	Maurivin; France; vineyard	Wine
AWRI2910*^a^*	Maurivin Elegance	Maurivin; Portugal	PdM
AWRI2911*^a^*	Maurivin EP2	Maurivin	Wine
AWRI2912*^a^*	Maurivin Primeur	Maurivin	Other
AWRI2913*^a^*	Maurivin Sauvignon	Maurivin; France	Wine
AWRI2914*^a^*	Maurivin UOA Maxithiol	Maurivin	Wine
AWRI2921*^a^*	Lalvin C	Lallemand; France	Wine
AWRI2927*^a^*	Lalvin R-HST	Lallemand; Austria	Wine
AWRI2928*^a^*	Enoferm RP15	Lallemand; USA; wine	Wine
AWRI2931*^a^*	Lalvin CLOS	Lallemand; Spain	Wine
AWRI2933*^a^*	Lalvin Barolo BRL97	Lallemand; Italy	Wine
S288C		Laboratory reference strain	Other
Sc Ancona Wine 28-AN		(Dunn *et al.* 2012)	Wine
Sc BrazFuel BG1		(Dunn *et al.* 2012)	Other
Sc BritAle NCYC1044		(Dunn *et al.* 2012)	Ale
Sc HefeAle W205		(Dunn *et al.* 2012)	Ale
Sc MoroccoBread G17		(Dunn *et al.* 2012)	Other
Sc SardCannonau 1446		(Dunn *et al.* 2012)	Wine
Sc SardSourdough S11		(Dunn *et al.* 2012)	Other
273614N		(Skelly *et al.* 2013)	Other
378604X		(Skelly *et al.* 2013)	Other
DBVPG6765		(Skelly *et al.* 2013)	Wine
SK1		(Skelly *et al.* 2013)	Other
UWOPS052272		(Skelly *et al.* 2013)	Other
YJM978		(Skelly *et al.* 2013)	Wine
YJM981		(Skelly *et al.* 2013)	Wine
YPS128		(Skelly *et al.* 2013)	Other
150A_Sc_DBVPG1106		(Bergström *et al.* 2014)	Wine
221A_Sc_L1528		(Bergström *et al.* 2014)	Wine
253AA_Sc_Y12		(Bergström *et al.* 2014)	Other
278A_Sc_UWOPS03-461.4		(Bergström *et al.* 2014)	Other
281_Sc_W303		(Bergström *et al.* 2014)	Other
308A_Sc_YJM975		(Bergström *et al.* 2014)	Wine
60A_Sc_DBVPG6044		(Bergström *et al.* 2014)	Other
91A_Sc_DBVPG1373		(Bergström *et al.* 2014)	wine
97A_Sc_Y55		(Bergström *et al.* 2014)	Other
WLP800*^a^*	Pilsner Lager	White Labs	Ale
WLP028*^a^*	Edinburgh Scottish Ale	White Labs	Ale
WLP001*^a^*	California Ale	White Labs	Ale
WLP023*^a^*	Burton Ale	White Labs	Ale
WLP002*^a^*	English Ale	White Labs	Ale
WLP004*^a^*	Irish Ale	White Labs	Ale
WY1084*^a^*	Irish Ale	WYeast	Ale
WLP013*^a^*	London Ale	White Labs	Ale
WLP500*^a^*	Monastery Ale	White Labs	Other
WLP775*^a^*	English Cider	White Labs	Wine
WLP705*^a^*	Sake	White Labs	Wine
WLP099*^a^*	Super High Gravity Ale	White labs	Wine
WLP862*^a^*	Cry Havoc	White Labs	Ale

a Commercial strains.

**Figure 1:**
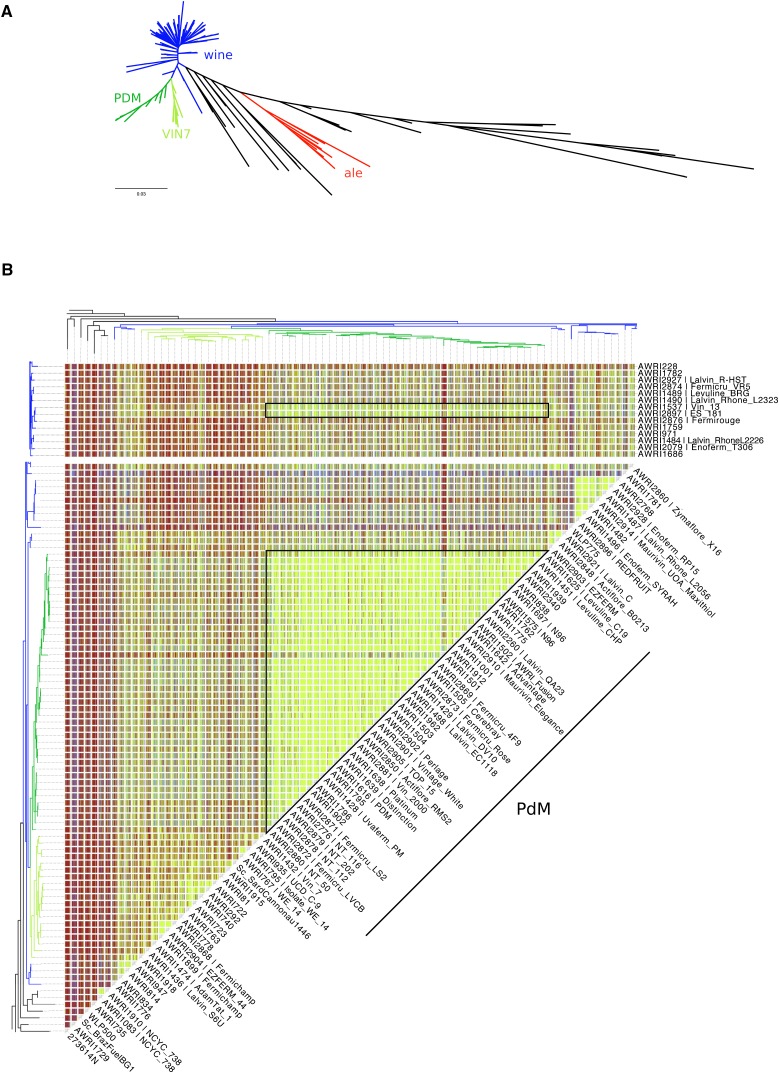
Genetic analysis of wine yeast strains. (A) An unrooted maximum-likelihood phylogeny of 236 strains of *S. cerevisiae*. Strains isolated from, or used in, ale brewing (red), or winemaking (blue and green) are highlighted. Dark- and light-green have been used to designate strains belonging to two main subclades within the wine yeasts. (B) Identity-by-state (IBS) analysis of the PdM clade and related strains (black boxes). SNPs were compared pairwise, across the collection of strains, with each variant position scored according to the pattern of nucleotide conservation. IBS state 2 (IBS2) represents identical diploid genotypes (*e.g.*, AA:AA, AT:AT), IBS1 loci share one allele (*e.g.*, AA:AT; AC:AT), while IBS0 loci are completely different (*e.g.*, AA:TT; AT:CG). Results were then smoothed using a 50-kb sliding window with a 25-kb step, with individual windows categorized according to four genomic states IBS2 (< 5% IBS1, <1% IBS0; green), IBS2|1 (≥ 5% IBS1, < 1% IBS0; blue), IBS2|1|0 (≥ 5% IBS1, ≥ 1% IBS0; red), and IBS2|0 (< 5% IBS1, ≥ 1% IBS0; red).

A whole-genome maximum-likelihood phylogeny was constructed based upon 1,455,253 bp of genome sequence, which exceeded coverage thresholds for SNP calling in all 236 strains (Figure 1). The resulting phylogeny displayed very clear stratification, with all but four of the commercial wine strains, and nine of the strains from the AWRI culture collection, clustering within a large, and highly related, clade containing other strains of either wine, or European origin, which is consistent with previous studies (Fay and Benavides 2005; Liti *et al.* 2009; Borneman *et al.* 2011; Dunn *et al.* 2012). This wine clade was characterized by little overall genetic variation, and the presence of a prominent subclade comprised of a third of all of the wine strains (Figure 1). This subclade could be further divided into two distinct lineages. The first, comprising the majority, was dominated by strains associated with the *Prize de Mousse* (PdM) collection of champagne yeasts, such as PDM, EC1118, and N96. The second lineage was dominated by other fructophillic strains, such as Fermichamp, and the *Saccharomyces* interspecific hybrids S6U and VIN7.

Given that the phylogeny may be confounded by attempts at breeding between strains (either natural or as part of a strain development program), IBS analysis was also employed in order to ascertain the pairwise level of relatedness for all 27,730 pairwise combinations of strains (Figure S1). This data reinforced the highly related nature of the PdM clade, with the majority of the group displaying almost identical genotypes, such that these strains could be considered to have arisen from a single progenitor strain, or highly interrelated progenitor population (Figure 1B). The exceptions to this are AWRI1501, NT116, NT202, and NT112, which all display a higher than average amount of IBS2|1 events, normally indicative of parent–progeny relationships. AWRI1501 is a 1*n*:1*n* *S. cerevisiae* × *S. paradoxus* interspecific hybrid, with the *S. cerevisiae* parent being a spore of AWRI838 (Bellon *et al.* 2015). The NT series of wine yeast are also the result of structured breeding, sharing a common PdM-series parent (N96). In addition to NT series strains that fell within the PdM clade, there was a higher degree of relatedness than expected from the structure of the phylogeny between the PdM clade and the strains AWRI1537 (Vin13) and AWRI2897 (ES 181). These two strains, which are adjacent on the phylogeny, display a pattern that, like the NT series, is consistent with these strains being hybrid progeny of a cross between a PdM-clade parent and a second, wine yeast, strain.

Like the wine clade, of the 13 commercially available “brewing” strains that were sequenced, the nine ale strains formed a clade that included other known ale isolates (Figure 1). Interestingly, three of the remaining strains (WLP705; sake, WLP099; high gravity ale, and WLP775; cider) were distributed throughout the wine yeast clade (Figure S1). These out-of-industry positions in the phylogeny were supported by the fact both WLP705 and WLP099 contain wine-specific maker loci, while lacking the pan-genomic hallmarks of true sake yeasts from the Far-East and ale-specific marker loci, respectively (Akao *et al.* 2011; Borneman *et al.* 2013) (Figure S2), and suggests that phenotypic spill-over can occur between industries for some strains.

### Evidence for interspecific hybridization

**Figure 2:**
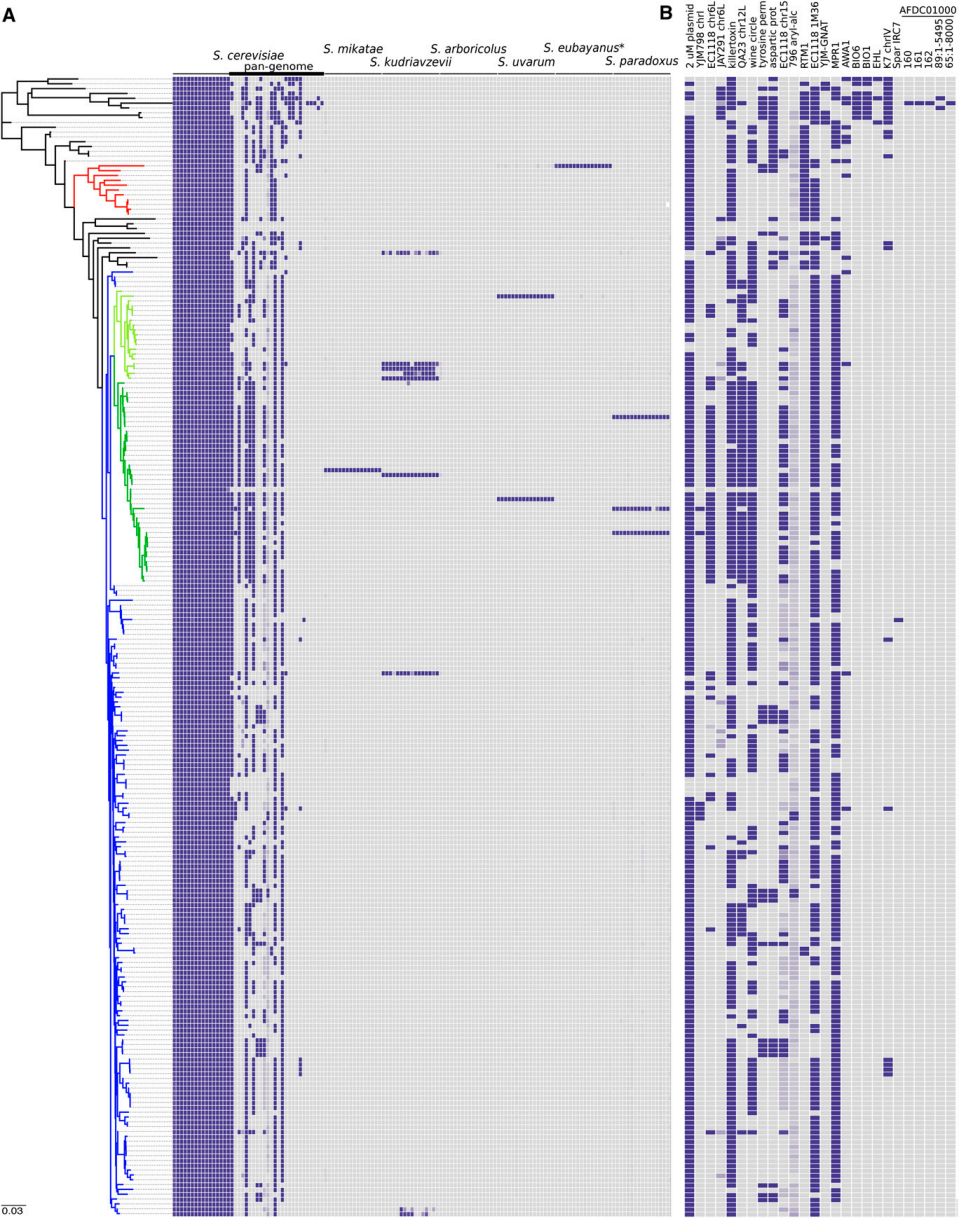
Genomic content differs across strains. (A) Sequence coverage was used to determine the genomic contribution of sequences from the *Saccharomyces sensu stricto* group in each strain. Each tile represents one of the 16 chromosomes of each species, except for the *S. cerevisiae* sequence set, which also contains 26 strain-variable accessory (pan-genome) loci. Strains are ordered according to the genome-wide SNP phylogeny, and colored as in Figure 1A. (B) A detailed display of the *S. cerevisiae* accessory elements of the pan-genome. Sequences of each locus can be found in File S1, and a high resolution figure, containing strain names is presented in Figure S2.

There are numerous examples of interspecific *Saccharomyces* hybrids from both brewing and winemaking environments, including the lager yeast, *S. pastorianus* (*S. cerevisiae* × *S. eubayanus*; (Dunn and Sherlock 2008; Nakao *et al.* 2009; Libkind *et al.* 2011) and some commercially available wine strains, such as VIN7 (*S. cerevisiae* × *S. kudriavzevii*; Bradbury *et al.* 2006; Borneman *et al.* 2012; Dunn *et al.* 2012), S6U (*S. cerevisiae* × *S. uvarum*; Naumova *et al.* 2005; Almeida *et al.* 2014; Pérez-Torrado *et al.* 2015), EP2 (*S. cerevisiae* × *S. kudriavzevii*; Dunn *et al.* 2012), and NT50 (*S. cerevisiae* × *S. kudriavzevii*; Bradbury *et al.* 2006; Dunn *et al.* 2012). All of the *S. cerevisiae* strains analyzed in this study were therefore interrogated for the presence of significant genomic contributions from other members of the *Saccharomyces sensu stricto* clade (Figure 2A).

**Figure 3:**
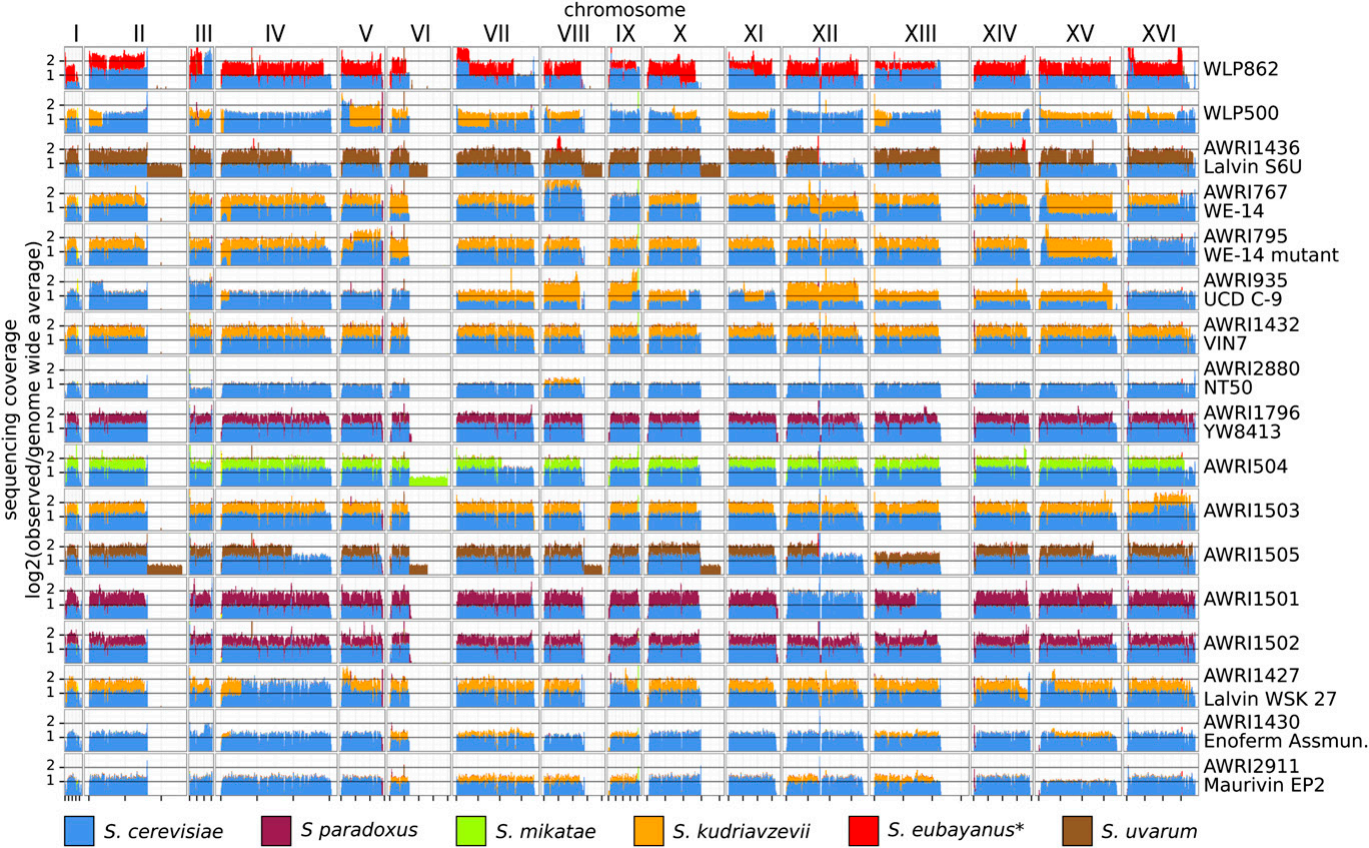
Inter-specific hybrids. Sequence coverage is displayed for strains that contained at least a 10% contribution from at least one chromosome from a non-*S. cerevisiae*. Coverage values are normalized to the genome wide average, with values color coded according to the donor species.

While the proportions of non-*S. cerevisiae* sequences were highly variable, but generally very low, 17 strains were found to contain greater than 10% of at least one chromosome of at least one other *Saccharomyces sensu stricto* species, with contributions from *S. kudriavzevii* (*n* = 10) being observed most frequently (Figure 2A). Of these 17, 12 appear to contain an almost complete, second non-*S. cerevisiae* genome (Figure 3). At least five of these (AWRI1501, AWRI1502, AWRI1503, AWRI1504, and AWRI1505), were laboratory generated via rare mating events between a wine strain of *S. cerevisiae* and other *Saccharomyces sensu stricto* members for the production of new commercial strains (Bellon *et al.* 2011, 2013, 2015). Of interest, despite being used as an ale-brewing yeast, the genome of WLP862 clearly classifies it as lager yeast (*S. pastorianus*) (Figure 3). The remaining strains all displayed highly variable levels of aneuploidy, with strain NT50, for example, predicted to comprise a tetraploid *S. cerevisiae* genome with only a single copy of *S. kudriavzevii* chromosome XIII. Furthermore, both S6U and WLP862 were shown to contain significant genomic contributions from three different species; however, the third species was shown to contribute a minor (∼10%) portion of its genome (Figure 3).

### Pan-genomic analysis

In addition to the strain-specific genomic contributions from *Saccharomyces* species other than *S. cerevisiae*, there were also significant intraspecific differences in several loci that have been shown to comprise the accessory, or noncore elements of the pan genome of *S. cerevisiae* (Figure 2B). Of these loci, two in particular, the “wine-circle” and the “*RTM1*-cluster,” broadly define strains used for winemaking and brewing, respectively (Borneman *et al.* 2011, 2013). Of the 124 strains found to carry the wine-circle, 111 (90%) are found within the generic wine clade; 35 strains were shown to carry the *RTM1*-cluster, of which 33 (94%), were from outside the wine clade. All 11 of the brewing strains carried the *RTM1*-cluster, but lacked the wine-circle. Interestingly, of the 13 strains from outside of the wine-clade that proved positive for the wine circle, 11 (85%) also contain the *RTM1*-cluster. At least four of these are commercial wine yeast strains, and the vast majority were highly heterozygous (see below). This suggests that they represent interclade hybrids between wine and nonwine parental strains that contain marker genes for both clades, and mosaic hybrids such as those commonly observed in natural populations (Liti *et al.* 2009; Hyma and Fay 2013; Clowers *et al.* 2015b).

Like the wine-circle, the yeast stress response gene *MPR1* (Shichiri *et al.* 2001) was also shown to be primarily associated with the wine-clade. Of the 197 strains that were shown to possess *MPR1*, 182 were within the wine clade (92% of the wine clade strains), with the remaining 15 in the nonwine group (38%). As for the wine-circle, all of the strains from within the ale subclade were shown to lack *MPR1*.

Finally, there were several loci, including the biotin-prototrophy genes *BIO1* and *BIO6* (Hall and Dietrich 2007), that were found almost exclusively in strains of Far-Eastern origin, such as those used for the production of sake. These genetic loci do not appear to have been introgressed into either brewing or winemaking strains, and may provide a source of useful unharnessed genetic variation for future wine yeast strain development.

Of the accessory loci that have previously been identified in wine strains, the aryl-alcohol cluster had been identified in only one strain: AWRI796 (Borneman *et al.* 2011). This current study identified another three strains in which this cluster is present: AWRI1494 (Enoferm M2), AWRI1483 (Lalvin ICV D254), and AWRI2255 (Uvaferm HPS). While Enoferm M2 and AWRI796 appear to be genomically equivalent (see below), Lalvin ICV D254 and Uvaferm HPS (which are also equivalent to each other) are distinct from this pair (> 2% IBS0 events), and display no evidence for recent common parentage. This suggests either multiple independent horizontal transfer events occurred with this rare accessory locus, or that the strains shared a past common ancestor, but any clear IBS signal for this event has been lost.

### Heterozygosity and genome renewal

**Figure 4:**
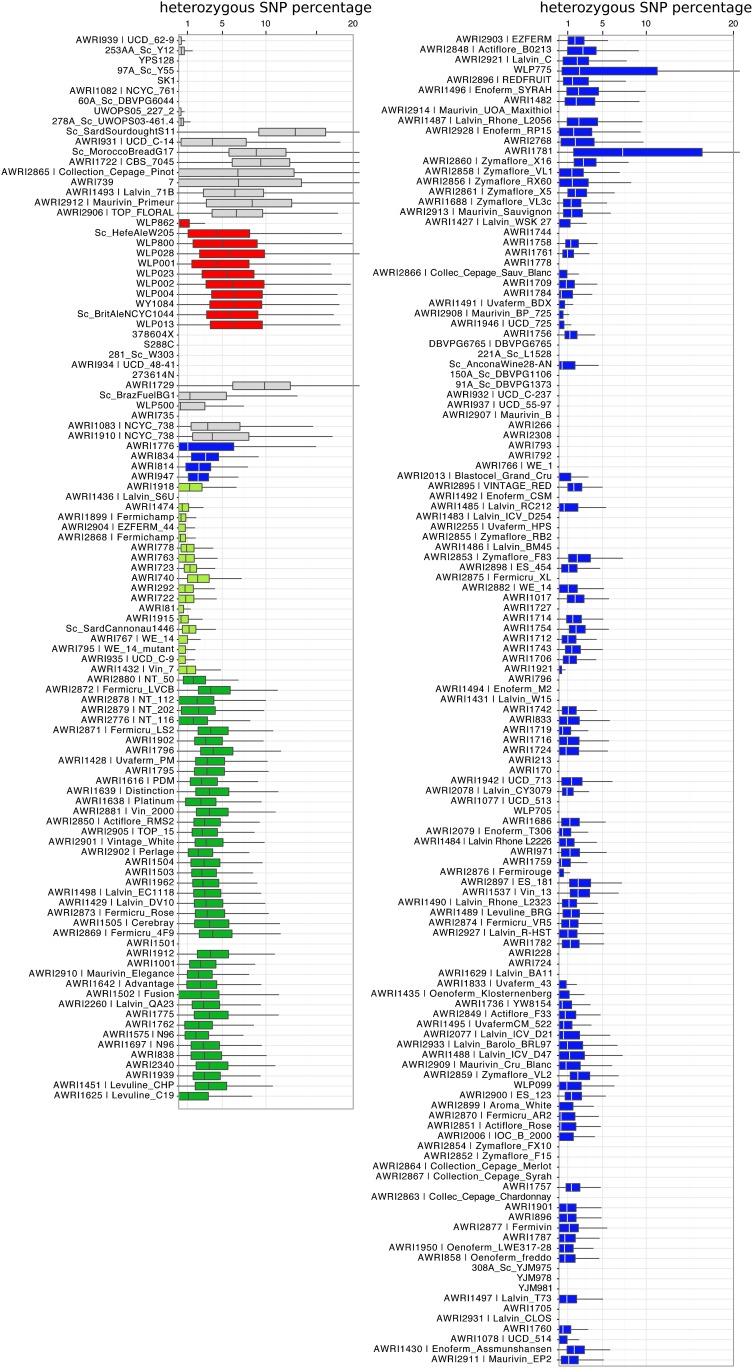
Heterozygosity is commonly observed in *S. cerevisiae* wine strains. Heterozygosity levels observed in 50-kb sliding windows (25 kb step) across the *S. cerevisiae* chromosomes (I–XVI) in each strain. Box and whisker plots summaries are shown. Median values are also listed for each strain. The plot for each strain is shaded according to the phylogenetic clades defined in Figure 1A.

As *S. cerevisiae* strains are generally able to undergo sporulation and mating type switching, the formation of homozygous diploids has been postulated to occur frequently in nature, leading to “genome renewal” (Mortimer *et al.* 1994; Bradbury *et al.* 2006). In order to determine the level of heterozygosity present in each strain, SNP calls made against S288c were classed as either homozygous or heterozygous based on the frequency of multiple alleles at each nucleotide position in the genome (a minimum frequency of 30% was required to call a heterozygous SNP). Data were then collected for 10 kb genomic windows (5 kb step), in which the proportion of heterozygous and homozygous SNPs were calculated (Figure 4). Levels of heterozygosity ranged considerably across the strains, but also displayed significant variation within the genome of individual strains, with evidence for large blocks of homozygosity present within otherwise heterozygous genomes (Figure S3). Using a genome-wide 0.75 quartile cut-off of zero heterozygous SNPs to class strains as homozygous (to account for false negative variant calls), 55 strains were considered to be homozygous, with 15 of these being the result of single-spore isolation prior to sequencing (Liti *et al.* 2009).

However, even when considering only commercial wine strains, 15% (16 of 106) are predicted to be homozygous. These homozygous strains are likely to be the products of sporulation and selfing (Mortimer *et al.* 1994), although there may be cases in which specific phenotypes have been introduced via backcrossing. This appears to be the case for AWRI2914 (Maurivin UOA Maxithiol)—a strain that appears genetically similar, albeit homozygous, to heterozygous diploid commercial strains such as AWRI1487 (Lalvin Rhone L2056) and AWRI2928 (Enoferm RP15), but which contains the Irc7 paralog from *S. paradoxus* (Roncoroni *et al.* 2011).

Of those strains displaying the highest levels of heterozygosity, the ale yeasts and baking strains figured prominently, which may be due to the common occurrence of polyploidy in these strains. Of the commercial wine yeasts, AWRI2912 (Maurivin Primeur), AWRI2865 (Collection Cepage Pinot), AWRI1493 (71B), and AWRI2906 (Top Floral), were shown to have the highest levels of heterozygosity, and were all located outside of the European wine yeast clade. As mentioned previously, it is likely that some of these strains have undergone recent interclade hybridization events, as all contain both the wine-circle and the ale-yeast *RTM1* cluster of marker genes (Figure 2C). Interestingly, there is some evidence that the trade off for the increased genetic diversity afforded by this interclade hybridization, is fermentation robustness, with 71B being considered less robust than most wine strains in some environments (Aranda *et al.* 2006; Schmidt *et al.* 2011).

### Genomic equivalency and strain redundancy

**Figure 5:**
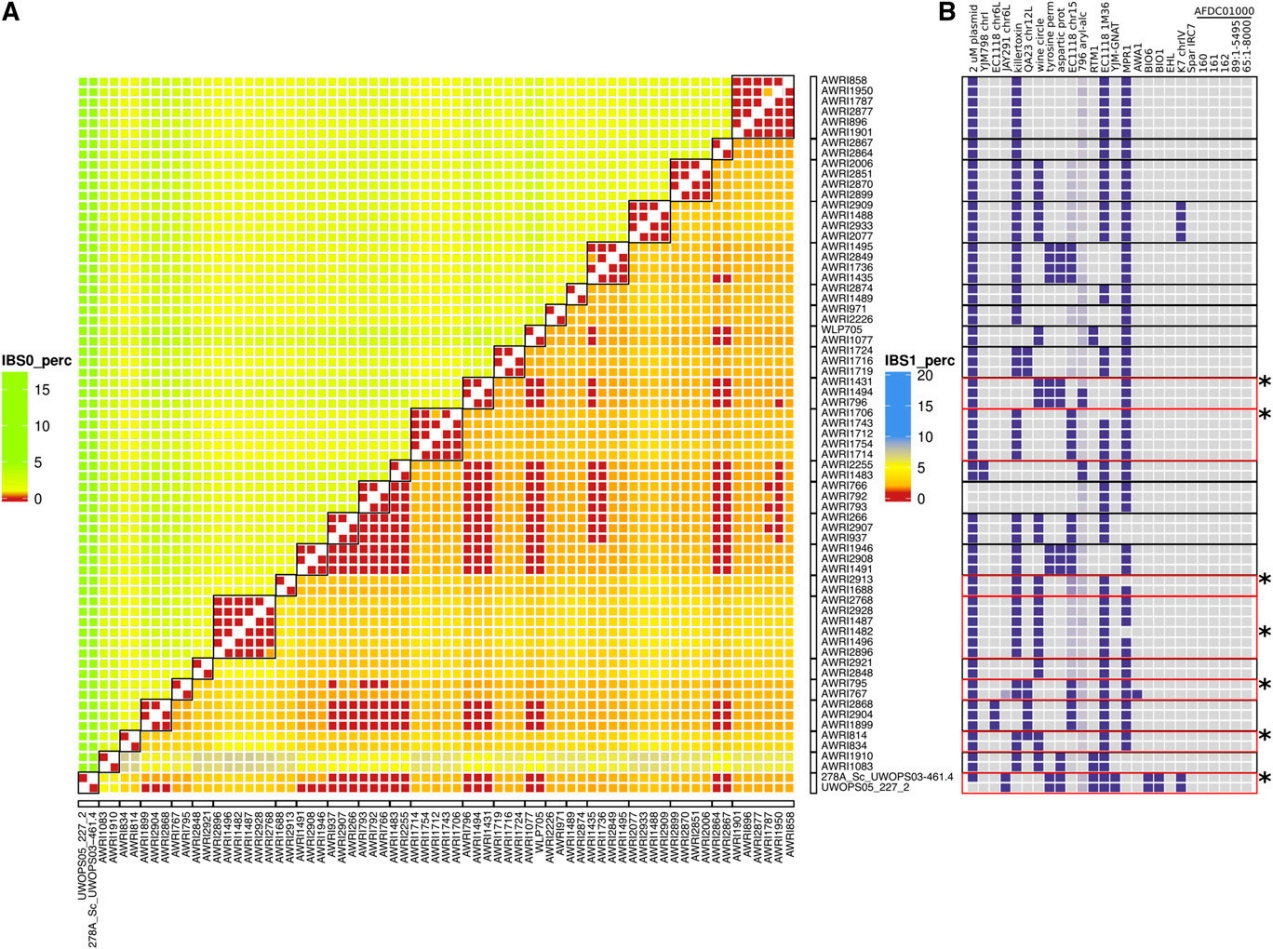
Genetic equivalence is common across wine yeast strains. (A) IBS2, IBS1, and IBS0 values were summed for each pair of strains, with IBS0:IBS2 and IBS1:IBS2 proportions calculated for each pair. Pairs displaying IBS0:IBS2 ≤ 0.05% and IBS1:IBS2 ≤ 1% were classified as being genetically equivalent at the SNP level. Groups of equivalent strains are bound by black boxes. (B) The composition of strain-specific pan genome in equivalent strain groups. Equivalence groups that contain variable accessory loci are boxed in red. The individual variable strain(s) are highlighted by asterisks.

From the combined genomic data available, it is apparent that, even if the large PdM clade is excluded, there are many yeast strains that appeared genetically identical. For some of these, this was due to multiple, independent isolates of the same strain being sequenced (*e.g.*, AWRI1083, NCYC 738 and AWRI1910, and NCYC 738), or the direct derivative of another strain being sequenced (AWRI767, WE 14 and AWRI795, and a spontaneous mutant of WE 14). Using these control comparisons as a baseline for false-positives in the SNP calling protocol, a baseline of 0.05% total IBS0, and 1% total IBS1 events between strains was chosen to reflect strains that show overall genetic equivalence (Figure S4). By applying these parameters, there were 69 strains that displayed genomic equivalence with at least one other strain (Figure 5). These could be further divided into 23 distinct equivalence groups, with the largest of these (two in total) being defined by six strains each, and with 13 groups containing at least two independent commercial isolates.

However, despite being genomically redundant at the level of SNP polymorphism, there were seven equivalency groups where one strain displayed a different pattern of accessory loci to the other member(s), with this generally involving a single accessory locus (Figure 5B). For example, the high throughput SNP pipeline showed that AWRI796, AWRI1494 (Enoferm M2), and AWRI1431 (Lalvin W15) differed only by up to 29 called heterozygous differences, yet Lalvin W15 lacks the entire 45 kb aryl-alcohol cluster (Figure 5). Likewise, strain AWRI1482, a member of the large equivalence group that includes the commercial strains AWRI1487 (Lalvin Rhone L2056) and AWRI2928 (Enoferm RP15), lacks the *MPR1* locus that is present in all other strains of this clade (Figure 5).

These differences in accessory loci, and the potential for small numbers of SNPs between otherwise redundant strains, likely reflect the variation that can arise during the independent isolation (and the possibility of long-term passaging) of new strains from “identical” progenitor material, or, in limited cases in this dataset, from the isolation of mutant strains from parental populations. The concerted loss of large tracts of DNA, such as the 45-kb aryl-alcohol cluster of AWRI796, supports the view of subtelomeric genomic plasticity leading to high rates of concerted gene gain and loss in these regions (Argueso *et al.* 2009).

### The PdM clade

**Figure 6:**
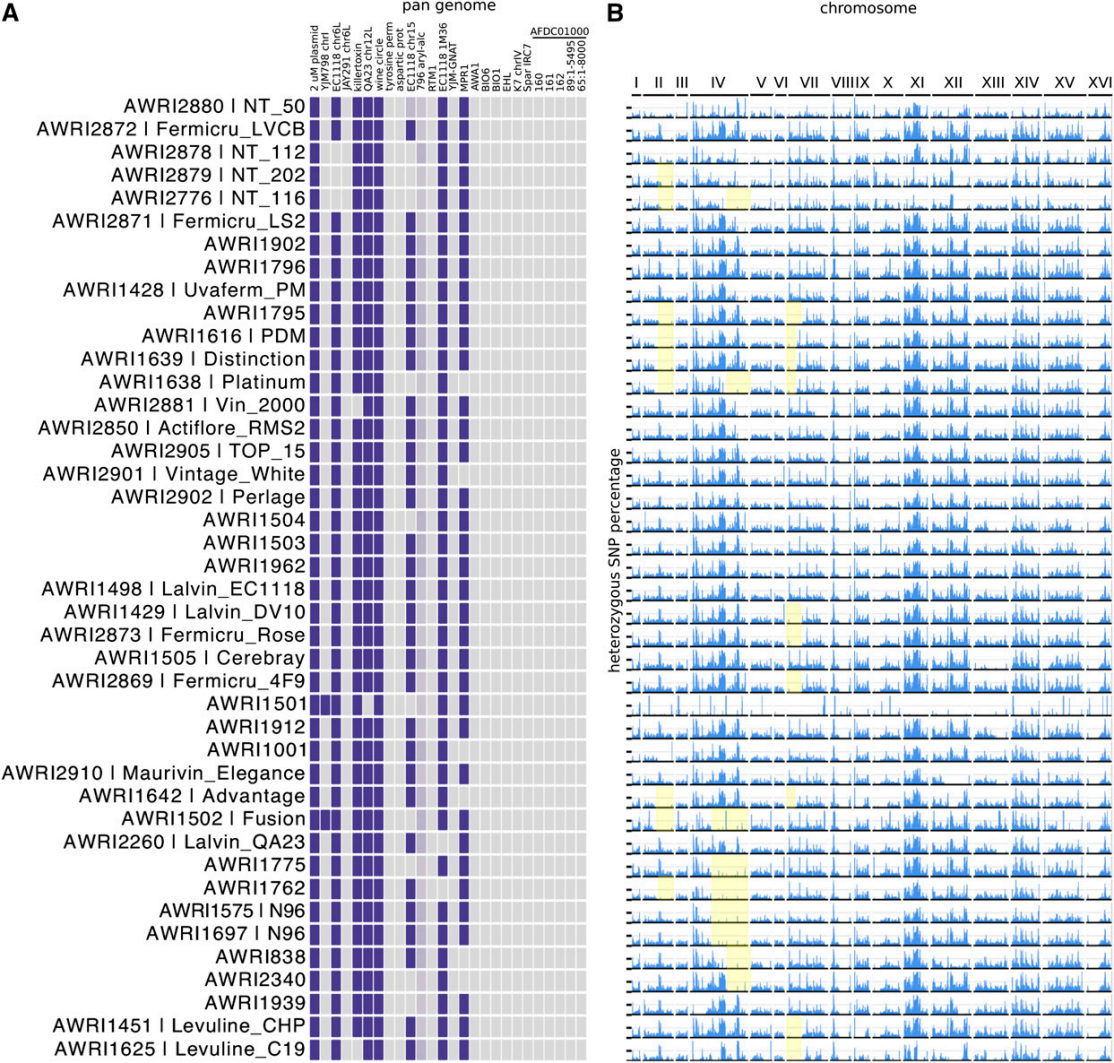
Genetic variation in the PdM clade of wine yeasts. (A) Pan genomic variation across the PdM clade. (B) Loss-of-heterozygosity (LOH) variation. The heterozygous SNP percentage is plotted as in Figure 4B, for the PdM clade. Three common regions of LOH in subsets of strains within the clade are highlighted in yellow.

Within the PdM clade, there were an additional 163 pairs of strains that passed the 0.05% total IBS0, and 1% total IBS1, test for genetic equivalency. Unlike the majority of the nonPdM strains, a continuum of values were observed (Figure S4), such that there were many more pairs that fell just outside of the threshold. This reinforces the fact that, while the PdM clade has a very recent common ancestry, the highly desirable winemaking traits of PdM yeasts have seen a wide variety of isolates and strain-development programs focusing on these strains. This has resulted in a large collection of similar, but not identical, strains, as highlighted by subtle heterozygosity and pan-genome differences (Figure 6). Several strains within the group have lost one or more accessory regions relative to the other members of the clade (Figure 6A), with 11 strains lacking the accessory locus that was first identified at the subtelomeric region of chromosome XV of EC1118 (Novo *et al.* 2009), and six strains lacking the *MPR1* stress resistance gene (Shichiri *et al.* 2001).

When heterozygosity patterns are examined, there are numerous examples of localized loss-of-heterozygosity (LOH) in members of the PdM clade, with some regions conserved across multiple isolates (Figure 6B). There is a characteristic LOH event that encompasses most of chromosome IV in N96 (AWRI 1575 and AWRI1697), as well as AWRI1775 and AWRI1762. A smaller LOH event in the same area is also found in AWRI838, AWRI2340, AWRI1638 (Platinum), and AWRI2776 (NT 116). Likewise, there is a conserved LOH on the right arm of chromosome II in at least nine isolates, and the left arm of chromosome VII in another ten strains.

These data point to LOH events, resulting in the loss of SNPs, but also potentially heterozygous subtelomeric accessory genes being a common occurrence across this large, conserved group of highly successful wine yeasts, with the concomitant phenotypic consequences of these large structural changes likely driving differences in their commercial performance.

### Conclusions

Despite sequencing a large number of wine strains of *S. cerevisiae*, including the majority of those that are commercially distributed, all appear to represent a highly inbred population that contains relatively little genetic variation compared to the global pool of *S. cerevisiae* diversity. Indeed, a large percentage of the strains analyzed in this study fall within one exceptionally related clade. Strain development efforts should therefore be focused on introgressing new variation, from outside of the wine yeast clade, into these economically important yeasts in order to increase the genetic, and therefore phenotypic, diversity that can be exploited in this industry.

## 

## Supplementary Material

Supplemental Material
