# Supplementary material for: Whole Genome Comparison Reveals High Levels of Inbreeding and Strain Redundancy Across the Spectrum of Commercial Wine Strains of Saccharomyces cerevisiae
Source: G3 (Bethesda). 2016 Feb 11;6(4):957–71. doi: 10.1534/g3.115.025692 (PMC4825664; doi:10.1534/g3.115.025692)
Supplement: Supplemental Material [file supp_6_4_957__index.html]

Supplemental Material 

# Whole Genome Comparison Reveals High Levels of Inbreeding and Strain Redundancy Across the Spectrum of Commercial Wine Strains of *Saccharomyces cerevisiae*

## Supplemental Material for Borneman *et al.*, 2016

**Files in this Data Supplement:**

- Figure S1 - Identity-by-state (IBS) analysis. (.pdf, 2650 KB)
- Figure S2 - Genomic content across strains. (.pdf, 3112 KB)
- Figure S3 - Heterozygosity in S. cerevisiae wine strains. (.pdf, 1073 KB)
- Figure S4 - Genetic equivalence in the sequenced strains of S. cerevisiae. (.pdf, 1136 KB)
- File S1 - Fasta file of *S. cerevisiae* strain-specific loci used for mapping of the pan genome. (.zip, 79 KB)
