# Supplementary material for: Whole Genome Comparison Reveals High Levels of Inbreeding and Strain Redundancy Across the Spectrum of Commercial Wine Strains of Saccharomyces cerevisiae
Source: G3 (Bethesda). 2016 Feb 11;6(4):957–71. doi: 10.1534/g3.115.025692 (PMC4825664; doi:10.1534/g3.115.025692)
Supplement: Supplemental Material [file supp_g3.115.025692_FigureS1.pdf]

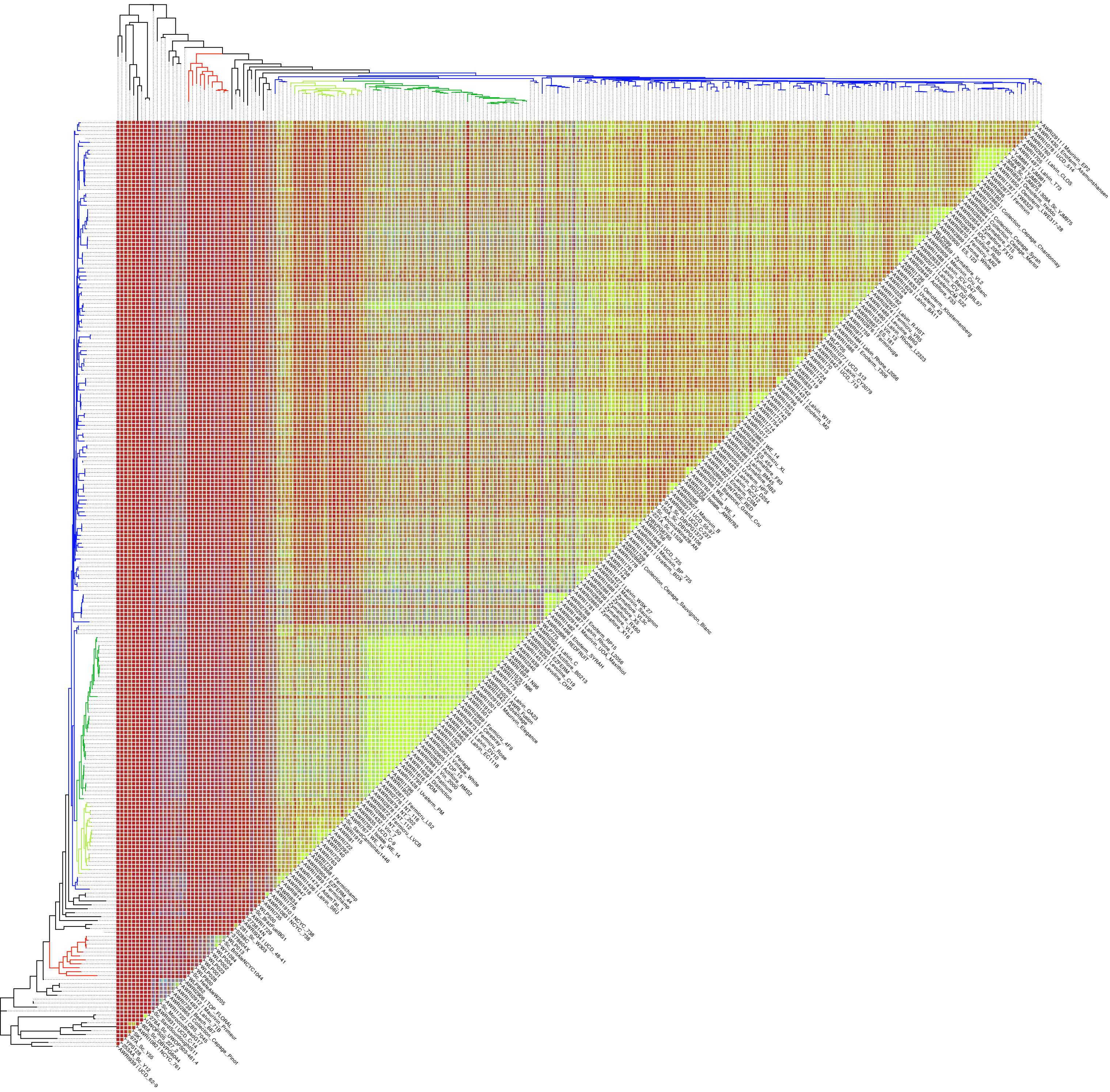

Figure S1. Identity-by-state (IBS) analysis. SNPs were compared pairwise, across the collection of strains, with each variant position scored according to the pattern of nucleotide conservation. IBS state 2 (IBS2) represents identical diploid genotypes (e.g. AA:AA, AT:AT), IBS1 loci share one allele (e.g. AA:AT; AC:AT) while IBS0 loci are completely different (e.g. AA:TT; AT:CG). Results were then smoothed using a 50 kb sliding window with a 25 kb step with individual windows categorized according to four genomic states IBS2 (<5% IBS1, <1% IBS0; green), IBS2|1 (5% IBS1, <1% IBS0; blue), IBS2|1|0 ((5% IBS1, 1% IBS0; red) and IBS2|0 ((<5% IBS1, 1% IBS0; red).
