# Supplementary material for: Whole Genome Comparison Reveals High Levels of Inbreeding and Strain Redundancy Across the Spectrum of Commercial Wine Strains of Saccharomyces cerevisiae
Source: G3 (Bethesda). 2016 Feb 11;6(4):957–71. doi: 10.1534/g3.115.025692 (PMC4825664; doi:10.1534/g3.115.025692)
Supplement: Supplemental Material [file supp_g3.115.025692_FigureS2.pdf]

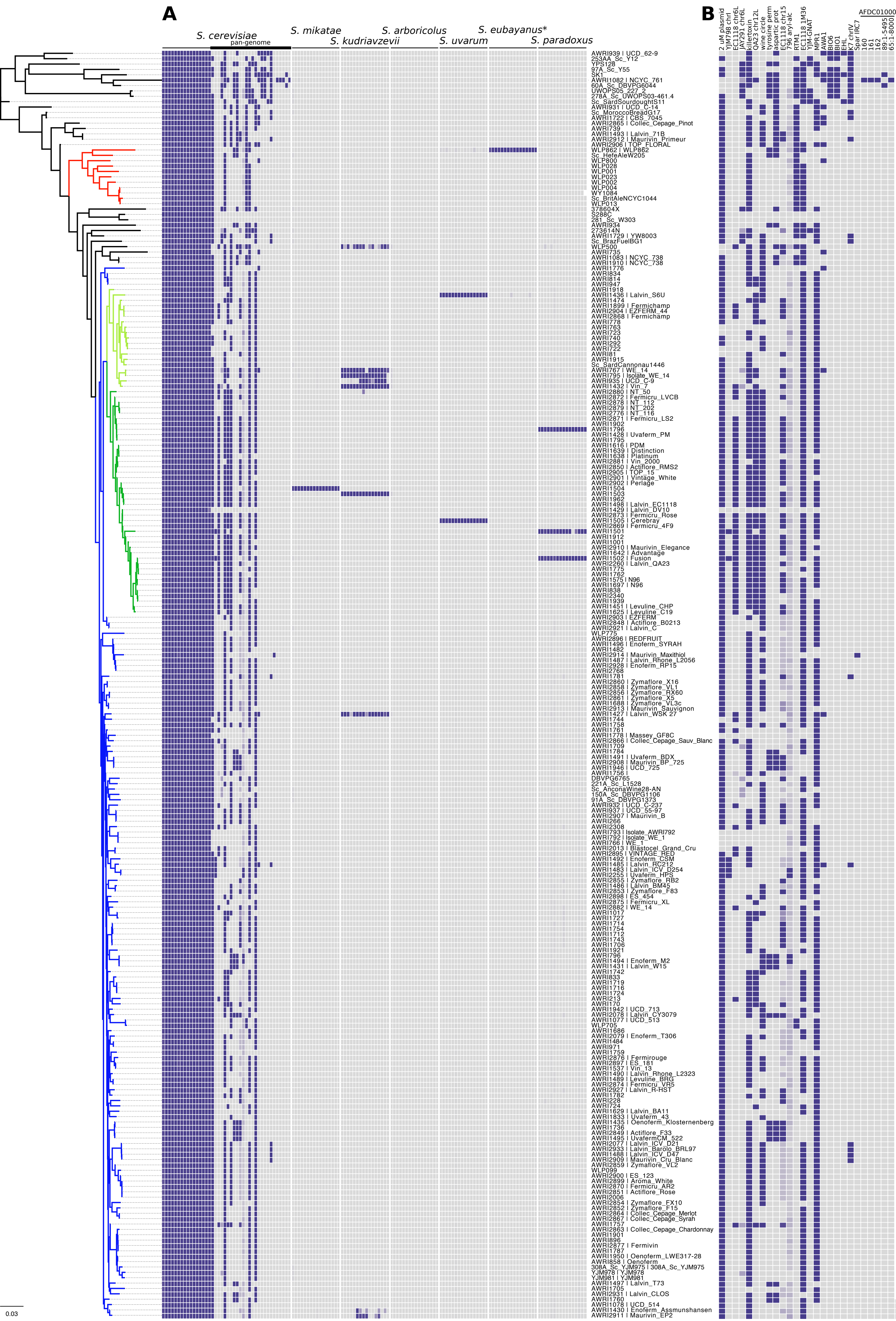

A
S. cerevisiae
pan-genome
S. mikatae
S. kudriavzevii
S. arboricolus
S. uvarum
S. eubayanus*
S. paradoxus
B
2 uM plasmid
YJM798 chrI
EC1118 chr6L
JAY291 chr6L
killertoxin
QA23 chr12L
wine circle
tyrosine perm
aspartic prot
EC1118 chr15
796 aryl-alc
RTM1
EC1118 1M36
YJM-GNAT
MPR1
AWA1
BIO6
BIO1
BHL
K7 chrIV
Spar IRC7
AFDC01000
160
161
162
89:1-5495
65:1-8000
0.03

Figure S2. Genomic content across strains. (A) Sequence coverage was used to determine the genomic contribution of sequences from the Saccharomyces sensu stricto group in each strain. Each tile represents one of the sixteen chromosomes of each species, except for the S. cerevisiae sequence set which also contains 26 strain-variable accessory (pan-genome) loci. Strains are ordered according to the genome-wide SNP phylogeny and colored according to clade (ale, red; wine, blue and green; PdM, dark green, VIN7, light green). (B) A detailed display of the S. cerevisiae accessory elements of the pan-genome.
