# Supplementary material for: Whole Genome Comparison Reveals High Levels of Inbreeding and Strain Redundancy Across the Spectrum of Commercial Wine Strains of Saccharomyces cerevisiae
Source: G3 (Bethesda). 2016 Feb 11;6(4):957–71. doi: 10.1534/g3.115.025692 (PMC4825664; doi:10.1534/g3.115.025692)
Supplement: Supplemental Material [file supp_g3.115.025692_FigureS3.pdf]

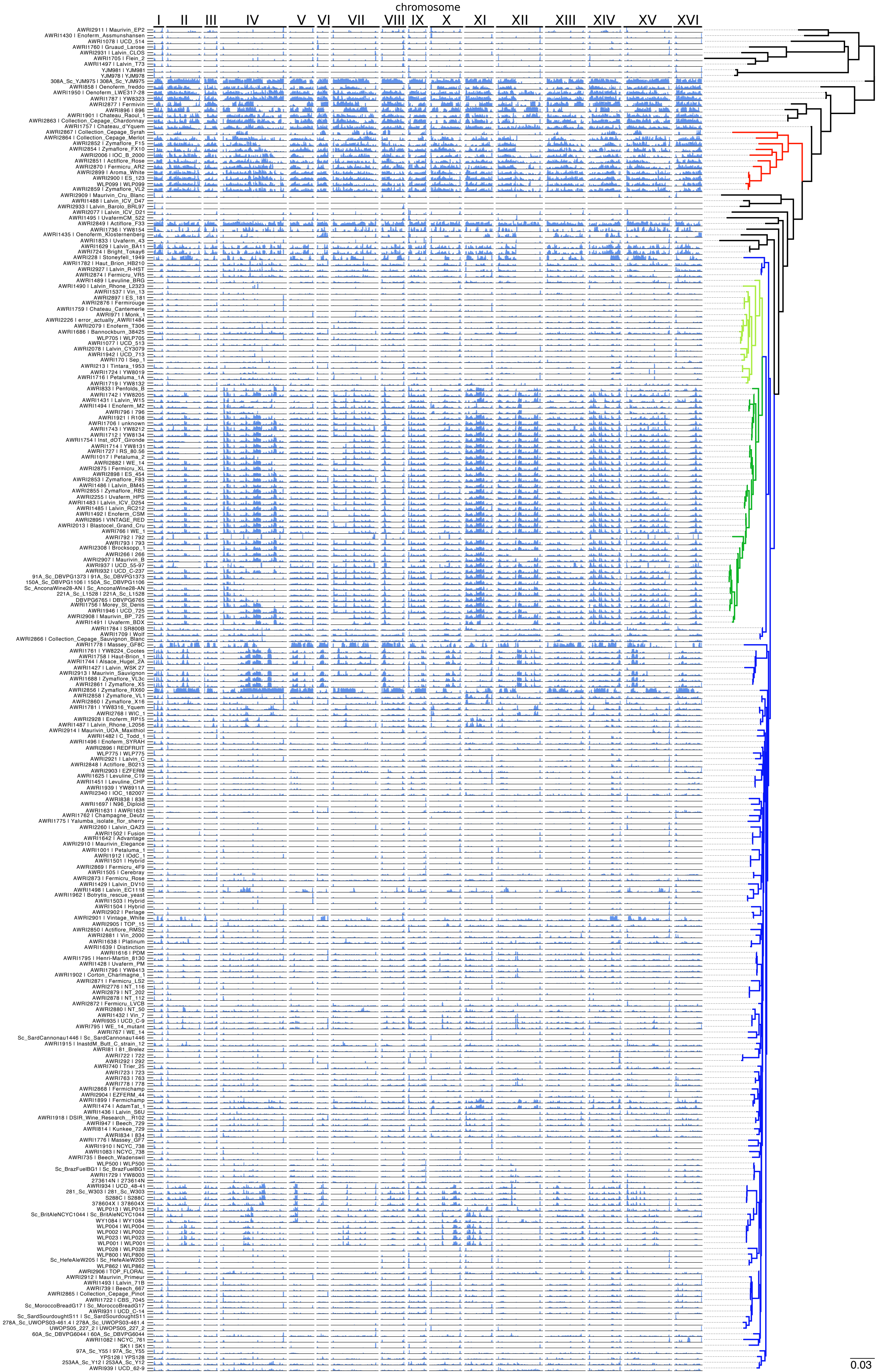

chromosome
I
II
III
IV
V
VI
VII
VIII
IX
X
XI
XII
XIII
XIV
XV
XVI
AWRI2911 | Maurivin_EP2
AWRI1430 | Enoferm_Assmunshansen
AWRI1078 | UCD_514
AWRI1760 | Gruaud_Larose
AWRI2931 | Lalvin_CLOS
AWRI1705 | Flein_2
AWRI1497 | Lalvin_T73
YJM981 | YJM981
YJM978 | YJM978
308A_Sc_YJM975 | 308A_Sc_YJM975
AWRI858 | Oenoferm_freddo
AWRI1950 | Oenoferm_LWE317-28
AWRI1787 | YW8323
AWRI2877 | Fermivin
AWRI896 | 896
AWRI1901 | Chateau_Raoul_1
AWRI2863 | Collection_Cepage_Chardonnay
AWRI1757 | Chateau_d'Yquem
AWRI2867 | Collection_Cepage_Syrah
AWRI2864 | Collection_Cepage_Merlot
AWRI2852 | Zymaflore_F15
AWRI2854 | Zymaflore_FX10
AWRI2006 | IOC_B_2000
AWRI2851 | Actiflore_Rose
AWRI2870 | Fermicru_AR2
AWRI2899 | Aroma_White
AWRI2900 | ES_123
WLP099 | WLP099
AWRI2859 | Zymaflore_VL2
AWRI2909 | Maurivin_Cru_Blanc
AWRI1488 | Lalvin_ICV_D47
AWRI2933 | Lalvin_Barolo_BRL97
AWRI2077 | Lalvin_ICV_D21
AWRI1495 | UvafermCM_522
AWRI2849 | Actiflore_F33
AWRI1736 | YW8154
AWRI1435 | Oenoferm_Klosterneuberg
AWRI1833 | Uvaferm_43
AWRI1629 | Lalvin_BA11
AWRI724 | Bright_Tokay6
AWRI228 | Stoneyfell_1949
AWRI1782 | Haut_Brion_HB210
AWRI2927 | Lalvin_R-HST
AWRI2874 | Fermicru_VR5
AWRI1489 | Levuline_BRG
AWRI1490 | Lalvin_Rhone_L2323
AWRI1537 | Vin_13
AWRI2897 | ES_181
AWRI2876 | Fermirouge
AWRI1759 | Chateau_Cantemerle
AWRI971 | Monk_1
AWRI2226 | error_actually_AWRI1484
AWRI2079 | Enoferm_T306
AWRI1686 | Bannockburn_38425
WLP705 | WLP705
AWRI1077 | UCD_513
AWRI2078 | Lalvin_CY3079
AWRI1942 | UCD_713
AWRI170 | Sep_1
AWRI213 | Tintara_1953
AWRI1724 | YW8019
AWRI1716 | Petaluma_1A
AWRI1719 | YW8132
AWRI833 | Penfolds_B
AWRI1742 | YW8205
AWRI1431 | Lalvin_W15
AWRI1494 | Enoferm_M2
AWRI796 | 796
AWRI1921 | R108
AWRI1706 | unknown
AWRI1743 | YW8212
AWRI1712 | YW8134
AWRI1754 | Inst_dOT_Gironde
AWRI1714 | YW8131
AWRI1727 | RS_80.56
AWRI1017 | Petaluma_2
AWRI2882 | WE_14
AWRI2875 | Fermicru_XL
AWRI2898 | ES_454
AWRI2853 | Zymaflore_F83
AWRI1486 | Lalvin_BM45
AWRI2855 | Zymaflore_RB2
AWRI2255 | Uvaferm_HPS
AWRI1483 | Lalvin_ICV_D254
AWRI1485 | Lalvin_RC212
AWRI1492 | Enoferm_CSM
AWRI2895 | VINTAGE_RED
AWRI2013 | Blastocel_Grand_Cru
AWRI766 | WE_1
AWRI792 | 792
AWRI793 | 793
AWRI2308 | Brocksopp_1
AWRI266 | 266
AWRI2907 | Maurivin_B
AWRI937 | UCD_55-97
AWRI932 | UCD_C-237
91A_Sc_DBVPG1373 | 91A_Sc_DBVPG1373
150A_Sc_DBVPG1106 | 150A_Sc_DBVPG1106
Sc_AnconaWine28-AN | Sc_AnconaWine28-AN
221A_Sc_L1528 | 221A_Sc_L1528
DBVPG6765 | DBVPG6765
AWRI1756 | Morey_St_Denis
AWRI1946 | UCD_725
AWRI2908 | Maurivin_BP_725
AWRI1491 | Uvaferm_BDX
AWRI1784 | SR800B
AWRI1709 | Wolf
AWRI2866 | Collection_Cepage_Sauvignon_Blanc
AWRI1778 | Massey_GF8C
AWRI1761 | YW8224_Cootes
AWRI1758 | Haut-Brion_1
AWRI1744 | Alsace_Hugel_2A
AWRI1427 | Lalvin_WSK 27
AWRI2913 | Maurivin_Sauvignon
AWRI1688 | Zymaflore_VL3c
AWRI2861 | Zymaflore_X5
AWRI2856 | Zymaflore_RX60
AWRI2858 | Zymaflore_VL1
AWRI2860 | Zymaflore_X16
AWRI1781 | YW8316_Yquem
AWRI2768 | WIC_1
AWRI2928 | Enoferm_RP15
AWRI1487 | Lalvin_Rhone_L2056
AWRI2914 | Maurivin_UOA_Maxithiol
AWRI1482 | C_Todd_1
AWRI1496 | Enoferm_SYRAH
AWRI2896 | REDFRUIT
WLP775 | WLP775
AWRI2921 | Lalvin_C
AWRI2848 | Actiflore_B0213
AWRI2903 | EZFERM
AWRI1625 | Levuline_C19
AWRI1451 | Levuline_CHP
AWRI1939 | YW8911A
AWRI2340 | IOC_182007
AWRI838 | 838
AWRI1697 | N96_Diploid
AWRI1631 | AWRI1631
AWRI1762 | Champagne_Deutz
AWRI1775 | Yalumba_isolate_flor_sherry
AWRI2260 | Lalvin_QA23
AWRI1502 | Fusion
AWRI1642 | Advantage
AWRI2910 | Maurivin_Elegance
AWRI1001 | Petaluma_1
AWRI1912 | IOdC_1
AWRI1501 | Hybrid
AWRI2869 | Fermicru_4F9
AWRI1505 | Cerebray
AWRI2873 | Fermicru_Rose
AWRI1429 | Lalvin_DV10
AWRI1498 | Lalvin_EC1118
AWRI1962 | Botrytis_rescue_yeast
AWRI1503 | Hybrid
AWRI1504 | Hybrid
AWRI2902 | Perlage
AWRI2901 | Vintage_White
AWRI2905 | TOP_15
AWRI2850 | Actiflore_RMS2
AWRI2881 | Vin_2000
AWRI1638 | Platinum
AWRI1639 | Distinction
AWRI1616 | PDM
AWRI1795 | Henri-Martin_8130
AWRI1428 | Uvaferm_PM
AWRI1796 | YW8413
AWRI1902 | Corton_Charlmagne_1
AWRI2871 | Fermicru_LS2
AWRI2776 | NT_116
AWRI2879 | NT_202
AWRI2878 | NT_112
AWRI2872 | Fermicru_LVCB
AWRI2880 | NT_50
AWRI1432 | Vin_7
AWRI935 | UCD_C-9
AWRI795 | WE_14_mutant
AWRI767 | WE_14
Sc_SardCannonau1446 | Sc_SardCannonau1446
AWRI1915 | InastdM_Butt_C_strain_12
AWRI81 | 81_Brelez
AWRI722 | 722
AWRI292 | 292
AWRI740 | Trier_25
AWRI723 | 723
AWRI763 | 763
AWRI778 | 778
AWRI2868 | Fermichamp
AWRI2904 | EZFERM_44
AWRI1899 | Fermichamp
AWRI1474 | AdamTat_1
AWRI1436 | Lalvin_S6U
AWRI1918 | DSIR_Wine_Research__R102
AWRI947 | Beech_729
AWRI814 | Kunkee_729
AWRI834 | 834
AWRI1776 | Massey_GF7
AWRI1910 | NCYC_738
AWRI1083 | NCYC_738
AWRI735 | Beech_Wadenswil
WLP500 | WLP500
Sc_BrazFuelBG1 | Sc_BrazFuelBG1
AWRI1729 | YW8003
273614N | 273614N
AWRI934 | UCD_48-41
281_Sc_W303 | 281_Sc_W303
S288C | S288C
378604X | 378604X
WLP013 | WLP013
Sc_BritAleNCYC1044 | Sc_BritAleNCYC1044
WY1084 | WY1084
WLP004 | WLP004
WLP002 | WLP002
WLP023 | WLP023
WLP001 | WLP001
WLP028 | WLP028
WLP800 | WLP800
Sc_HefeAleW205 | Sc_HefeAleW205
WLP862 | WLP862
AWRI2906 | TOP_FLORAL
AWRI2912 | Maurivin_Primeur
AWRI1493 | Lalvin_71B
AWRI739 | Beech_667
AWRI2865 | Collection_Cepage_Pinot
AWRI1722 | CBS_7045
Sc_MoroccoBreadG17 | Sc_MoroccoBreadG17
AWRI931 | UCD_C-14
Sc_SardSourdoughtS11 | Sc_SardSourdoughtS11
278A_Sc_UWOPS03-461.4 | 278A_Sc_UWOPS03-461.4
UWOPS05_227_2 | UWOPS05_227_2
60A_Sc_DBVPG6044 | 60A_Sc_DBVPG6044
AWRI1082 | NCYC_761
SK1 | SK1
97A_Sc_Y55 | 97A_Sc_Y55
YPS128 | YPS128
253AA_Sc_Y12 | 253AA_Sc_Y12
AWRI939 | UCD_62-9
0.03

Figure S3. Heterozygosity in S. cerevisiae wine strains. Heterozygosity levels observed in 50 kb sliding windows (25 kb step) across the S. cerevisiae chromosomes (I - XVI) in each strain. Strains are ordered according to the genome-wide SNP phylogeny and colored according to clade (ale, red; wine, blue and green; PdM, dark green, VIN7, light green).
