# Supplementary material for: Whole Genome Comparison Reveals High Levels of Inbreeding and Strain Redundancy Across the Spectrum of Commercial Wine Strains of Saccharomyces cerevisiae
Source: G3 (Bethesda). 2016 Feb 11;6(4):957–71. doi: 10.1534/g3.115.025692 (PMC4825664; doi:10.1534/g3.115.025692)
Supplement: Supplemental Material [file supp_g3.115.025692_FigureS4.pdf]

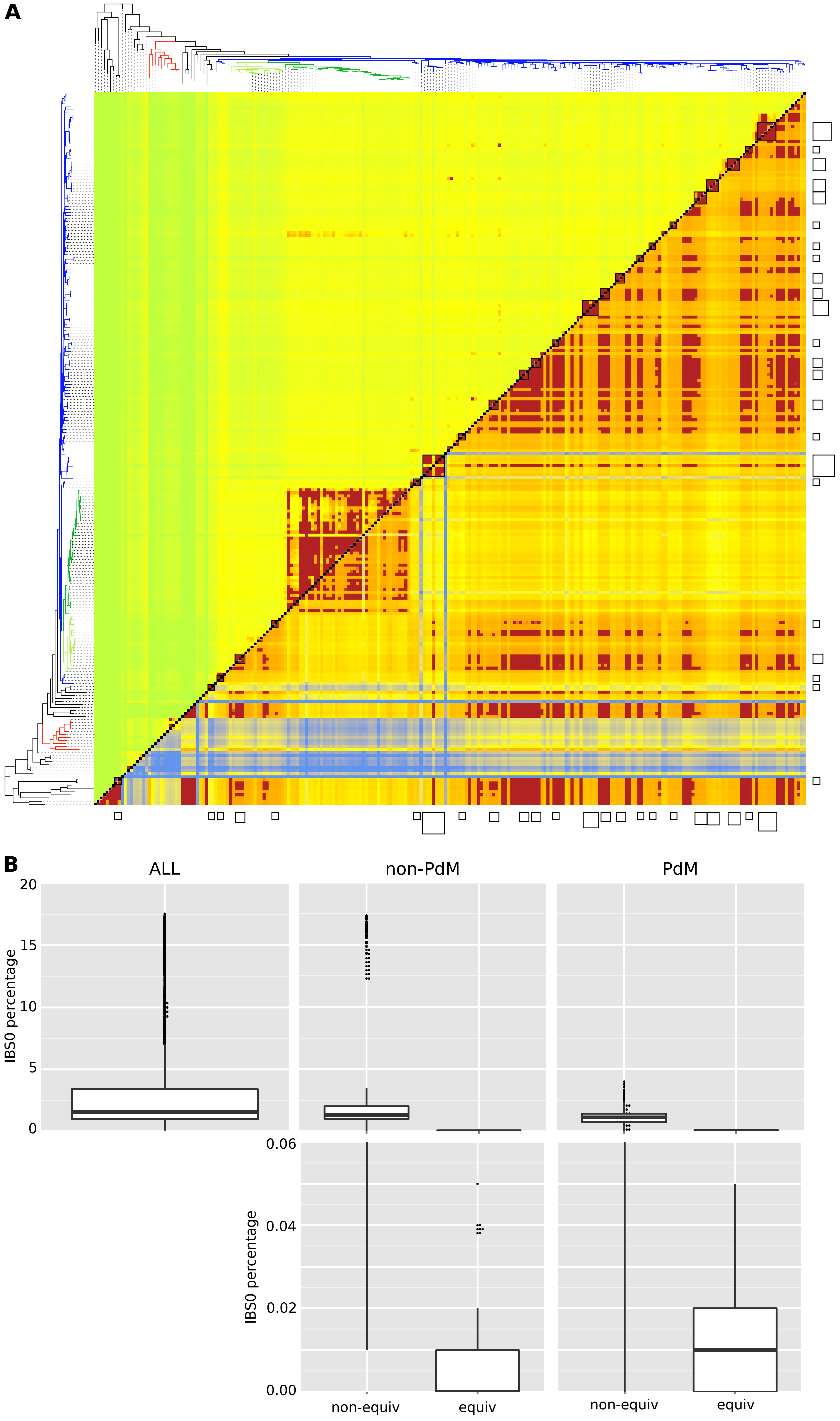
A
B
ALL
non-PdM
PdM
IBS0 percentage
20
15
10
5
0
0.06
0.04
0.02
0.00
non-equiv
equiv

Figure S4. Genetic equivalence in the sequenced strains of S. cerevisiae. (A) IBS2, IBS1 and IBS0 values were summed for each pair of strains, with IBS0:IBS2 and IBS1:IBS2 proportions calculated for each pair. Pairs displaying an IBS0:IBS2 $\leq$ 0.05% and an IBS1:IBS2 $\leq$ 1% were classified as being genetically equivalent at the SNP level. Groups of equivalent strains are bound by black boxes. (B) Box and whisker plots of the IBS0 percentage of specific groups of strains. The non-PdM and PdM clade strains are further divided into those that were classified as equivalent or non-equivalent. The PdM clade displayed far higher variance, with many strains at or near the 0.05% threshold compared to the non-PdM group.
